# The Relationship Between Fragility Fractures and Pain Experience: A Systematic Review

**DOI:** 10.3389/fmed.2021.609318

**Published:** 2021-05-24

**Authors:** Pei-En Chen, Ching-Wen Chien, Tao-Hsin Tung

**Affiliations:** ^1^Institute of Health Policy and Management, College of Public Health, National Taiwan University, Taipei, Taiwan; ^2^Taiwan Association of Health Industry Management and Development, Taipei, Taiwan; ^3^Institute for Hospital Management, Tsing Hua University, Shenzhen, China; ^4^Evidence-Based Medicine Center, Taizhou Hospital of Zhejiang Province Affiliated to Wenzhou Medical University, Linhai, China

**Keywords:** fragility fracture, pain, systematic review, fracture, discomfort

## Abstract

**Purpose:** This systematic review is conducted to explore the relationship between fragility fractures and pain experience.

**Methods:** We searched for relevant studies on Pubmed, Embase, Web of Science, and the Cochrane library without restrictions on language from inception until February 4th, 2021. The risk of bias and methodological quality was evaluated using the Newcastle-Ottawa Scale and ROBINS-I tool.

**Results:** Twenty-one studies were included in this systematic review. The so-called study reported participants with continuous post-fracture pain. The included studies showed that post- fractured pain can decrease with time, however, the continual pain can last at least 1 year even longer, and some participants would need to self-manage pain. Moreover, the limited range of motion was considered as a factor that might distress the normal development of daily activities.

**Conclusions:** The current evidence could not fully support that pain continues to influence patients' lives after a fragility fracture. However, it still showed the pain might come with fracture. The findings also could be useful to help health care providers better recognize and manage this clinical consequence of fractures. Nonetheless, future large-scale longitudinal studies will be required to evaluate the long-term effects of pain in fragility fractures.

## Introduction

The World Health Organization located osteoporosis at the primary health care level, reporting that “*a fracture caused by injury that would be insufficient to fracture a normal bone.the result of reduced compressive and/or torsional strength of bone”* (1). From a clinical perspective, fragility fractures are considered as skeletal complications, leading to substantial morbidity, longer hospitalization period, higher health care costs, poorest quality of life, more severe disabilities, and death (2). Different fracture locations may as well involve diverse symptoms across time. Hip fractures are conceived as the most serious kind; with a 1-year mortality rate of 21% for women and 31% for men (3). Other kind of fragility-related fractures at other anatomical locations has been related with lower quality conditions of life, although most studies tend to focus on the impacts of the hip or the vertebral fractures (4).

While significant improvements have been achieved— both in surgical procedures and treatment tools—in this area, current information on incidences, risk factors, and medical costs of pain appears to be highly restricted. Currently, pain assessment and relief for patients with fragility fractures before and after surgery are placed as crucial topics for research. Besides, as surgical indications tend to be a direct procedure for most patients suffering from this kind of fragility fracture and adequate anesthesia is the basis for these successful so-called surgical procedures, there is still room for improvement in various anesthesia and sedation techniques. A part of the patients with fragility fractures, however, show surgical contraindications or prefer conservative treatment; in this kind of situation it becomes, indeed, highly significant to use methods to help to relieve this related discomfort, reduce the risk of adverse effects and improve the overall quality of life, accurate diagnosis and efficient pain eradication.

The purpose of this study was to assess the relationship between fragility fractures and pain experience. The results from this systematic review could further understand the fragility of fractures related to pain and guide health care to address the issues which matter to such patients.

## Materials and Methods

The methodology was written based on several studies published (5–7).

### Literature Review

The Pubmed, Embase, Web of Science, and the Cochrane library for relevant studies without language limitations were used from inception until February 04, 2021. These databases included most of the academic research articles on this topic. The searched eligible studies were identified by scanning electronic databases using various combinations of Medical Subject Headings (MeSH) and non-MeSH terms.

### Data Sources and Search Methods

The search process was extended by (1) perusing the reference section of all relevant studies, and (2) manually searching through the abstracts of key journals and articles published at major annual meetings. The review's population, intervention, comparison, outcome (PICO) items defined the search strategy: Population: all population, Intervention: fracture, Comparison: not applicable; Outcome: Pain. The search terms included all field and the following: (fragility OR fracture OR low traumatic) AND (fracture OR break OR split OR crack) AND (Pain OR Long-term pain OR Painful OR suffer OR discomfort OR hurt OR irritation OR tenderness OR soreness OR Fracture-related limitations OR disability Or disabled). Table 1 shows the search strategy of databases.

**Table 1:** Search strategy in PubMed up till Febuary 4th, 2021.

**Pubmed**		***N***
#1	fragility [all field]	19,914
#2	fragile [all field]	22,727
#3	low traumatic [all field]	12,625
#4	#1 OR #2 O #3	53,368
#5	fracture [all field]	322,337
#6	break [all field]	119,498
#7	split [all field]	102,207
#8	crack [all field]	20,423
#9	#5 OR #6 OR #7 OR #8	554,762
#10	Pain [all field]	869,082
#11	Long-term pain [all field]	45,413
#12	Painful [all field]	900,316
#13	Suffer [all field]	1,307,321
#14	discomfort [all field]	48,254
#15	hurt [all field]	4,855
#16	irritation [all field]	52,785
#17	tenderness [all field]	24,481
#18	soreness [all field]	3,526
#19	Fracture-related limitations [all field]	85
#20	disability [all field]	374,533
#21	disabled [all field]	374,533
#22	#10 OR #11 OR #12 OR #13 OR #14 OR #15 OR #16 OR #17 OR #18 OR #19 OR #20 OR #21	1,740,707
#23	#4 AND #9 AND #22	1,479
**Cochrane**		***N***
#1	fragility [all field]	1,454
#2	fragile [all field]	736
#3	low traumatic [all field]	2,223
#4	#1 OR #2 O #3	4,313
#5	fracture [all field]	18,713
#6	break [all field]	4,066
#7	split [all field]	10,282
#8	crack [all field]	390
#9	#5 OR #6 OR #7 OR #8	32,832
#10	Pain [all field]	191,678
#11	Long-term pain [all field]	13,367
#12	Painful [all field]	12,200
#13	Suffer [all field]	7,294
#14	discomfort [all field]	16,901
#15	hurt [all field]	519
#16	irritation [all field]	5,867
#17	tenderness [all field]	3,618
#18	soreness [all field]	1,995
#19	Fracture-related limitations [all field]	10
#20	disability [all field]	37,247
#21	disabled [all field]	3,175
#22	#10 OR #11 OR #12 OR #13 OR #14 OR #15 OR #16 OR #17 OR #18 OR #19 OR #20 OR #21	234,933
#23	#4 AND #9 AND #22	745
**Embase**		***N***
#1	fragility [all field]	41,107
#2	fragile [all field]	29,980
#3	low traumatic [all field]	144
#4	#1 OR #2 O #3	69,324
#5	fracture [all field]	396,777
#6	break [all field]	72,142
#7	split [all field]	91,222
#8	crack [all field]	9,824
#9	#5 OR #6 OR #7 OR #8	563,937
#10	Pain [all field]	1,378,467
#11	Long-term pain [all field]	1,777
#12	Painful [all field]	90,626
#13	Suffer [all field]	98,951
#14	discomfort [all field]	84,393
#15	hurt [all field]	6,051
#16	irritation [all field]	48,794
#17	tenderness [all field]	37,726
#18	soreness [all field]	4,539
#19	Fracture-related limitations [all field]	1
#20	disability [all field]	334,924
#21	disabled [all field]	60,695
#22	#10 OR #11 OR #12 OR #13 OR #14 OR #15 OR #16 OR #17 OR #18 OR #19 OR #20 OR #21	1,916,555
#23	#4 AND #9 AND #22	4,262
**Web of Science**		***N***
#1	fragility [all field]	27,390
#2	fragile [all field]	40,871
#3	low traumatic [all field]	24,839
#4	#1 OR #2 O #3	91,094
#5	fracture [all field]	509,008
#6	break [all field]	407,261
#7	split [all field]	287,946
#8	crack [all field]	261,946
#9	#5 OR #6 OR #7 OR #8	1,351,490
#10	Pain [all field]	708,471
#11	Long-term pain [all field]	42,587
#12	Painful [all field]	53,923
#13	Suffer [all field]	349,061
#14	discomfort [all field]	44,770
#15	hurt [all field]	12,487
#16	irritation [all field]	21,493
#17	tenderness [all field]	16,422
#18	soreness [all field]	3,980
#19	Fracture-related limitations [all field]	27
#20	disability [all field]	285,912
#21	disabled [all field]	57,642
#22	#10 OR #11 OR #12 OR #13 OR #14 OR #15 OR #16 OR #17 OR #18 OR #19 OR #20 OR #21	1,399,557
#23	#4 AND #9 AND #22	1,764

### Data Extraction and Quality Assessment

A data extraction form was used to obtain the following data from the included studies: first author (publication year), country, study duration, study subjects, age of study subjects, sex, assigned groups, and outcomes. The abstract and full-text screening was undertaken by Pei-En Chen and Tao-Hsin Tung. An assessment of methodological quality was performed independently by the authors (Pei-En Chen and Tao-Hsin Tung). The Newcastle-Ottawa Scale (NOS) was applied independently by two authors to determine the consistency of the selected studies (6). Any disagreement was discussed with a third senior author (Ching-Wen Chien). The NOS applies three domains (selection of study groups, comparability, and outcome assessment) to assess the quality of studies. A study could be awarded up to one star for each item within the selection and outcome domains and up to two stars for comparability. We viewed it as a study of high quality if seven or more stars were awarded. Besides, to increase the reproducibility and comparability of this systematic review to future reviews on a similar topic, we also evaluated risk of bias assessment using Risk of Bias in Non-randomized studies of Interventions (ROBINS-I) due to since it is the newest and most robust method of identifying the risk of bias in systematic reviews and meta-analyses (7).

### Data Synthesis

The outcomes of the selected studies were assessed focusing on various measurements.

Follow by characteristics of outcome measurement:

1. Von Kroff questionnaire (which points both pain intensity score and disability score): It was developed in order to grade the severity of chronic pain (8).
2. EQ-5D (pain/discomfort): It is a standardized tool used to assess general health problems, which covers 5 main domains such as mobility, self-care, daily activities, pain/discomfort, and anxiety/depression (9). Thoughout this present research, only domains related to pain/discomfort will be discussed.
3. Numeric rating scale (NRS): Which displays results verabally reported by patients. The scale ranges from 0 (no pain) to 10 (worst imaginable pain) (10).
4. Visual Analog Scale (VAS, 0–10): Scale used in order to quantify a trait or attitude that is assumed to extend across a spectrum of values and cannot be directly measured easily. It is also used to measure the severity or frequency of different symptoms through epidemiological and clinical research (10).
5. SF-36 (pain/discomfort), which consists of an eight scaled score, containing weighted sums of questions (0–100). The eight sections are: vitality, physical functioning, bodily pain, general health perceptions, physical role functioning, emotional role functioning, social role functioning, mental health (10).
6. The Quality of Life Questionnaire (QUALEFFO-41; pain domain): To investigate about the improvements in the quality of life associated to day-to-day living, general well-being, and specific well-being of patients who have had any kind of the vertebral fractures named by International Osteoporosis Foundation (IOF) (11).
7. Geriatric Pain Scale (GPS, 0–100): Used to classify pain in patients and to assess physical, mental, cognitive, and behavioral responses to pain (12).
8. Pain Regulation Questionnaire (PRQ) which includes competences, intensity, anxiety, depression, avoidance, withdrawal and distraction of pain (13).
9. The amounts of individuals reporting pain.

The ROBIS tool was applied to assess the risk of bias in this systematic review. This tool consists of three phases; and this systematic review more specifically evaluated phase 2 and phase 3. During phase 2 there were four aspects evaluated: study eligibility criteria, identification and selection of studies, data collection and study appraisal, and synthesis and findings. Moreover, phase 3 integrated the overall risk from phase 2 (14).

### Statistical Analysis

The tool Review Manager 5.3 (The Nordic Cochrane Center, The Cochrane Collaboration, 2014) was used in this study. We presented the risk of pain as OR with 95% CI and assessed heterogeneity by using the *I*^2^ statistic. The *I*^2^ statistic is used to evaluate the degree of variation across studies due to heterogeneity rather than by chance alone (15).

## Results

### Study Characteristics

**Figure 1:**
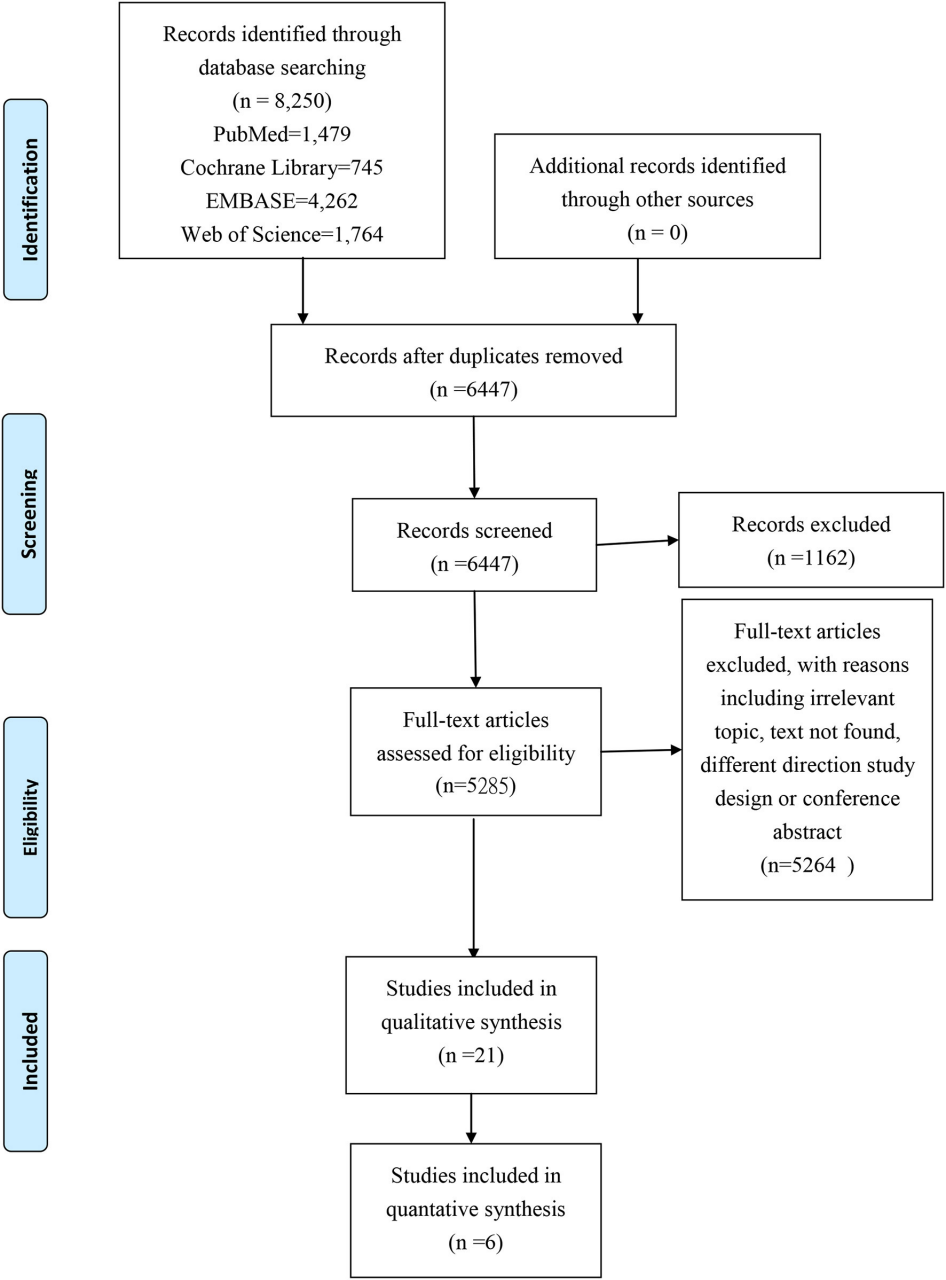
Prisma study flow chart.

Figure 1 illustrates the results of this systematic review. From all the databases we searched in, with 8,250 records collected and after removing 1,803 duplicate articles, there was a total of 1,162 records which were excluded due to is protocol or other conference abstracts. Five thousand two hundred sixty-four full-text articles were also excluded for reasons such as irrelevancy of the topic, incapability to find the related text, differences on the purposes on the study design, conferences abstracts, or fracture-pain related articles with drugs/treatment. Finally, following a thorough review of all candidate papers, we identified a total of 21 studies that addressed the relationship between fragility fractures and pain experience.

### Fragility Fracture and Pain Experience

#### Assessment of Pain and Disability

**Figure 2:**
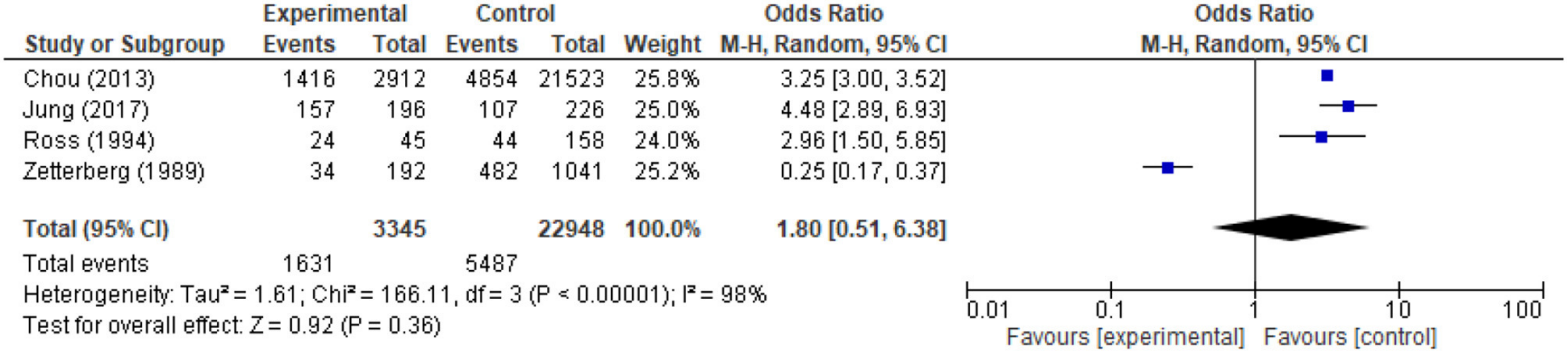
Odds of pain in patients with fracture.

**Figure 3:**
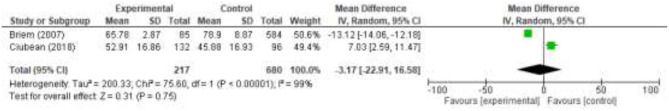
Mean difference of pain in patients with fracture: SF-36 (pain/discomfort domain).

In Chou et al. (16), Jung et al. (17), and Ross et al. (18) fracture group showed a higher frequency on reported pain. However, Zetterberg et al. (19) indicated a different tendency from the other 3 studies. Figure 2 displays that pain was not significantly associated with fracture than the control group. Figure 3 specifies that pain was not related to the fracture group (*p* = 0.75). Among the 21 included studies, there were 8 which investigated the risk for fragility fracture and pain in postmenopausal females and 3 in the elderly, due to its higher vulnerability.

Apart from the above mentioned studies, other studies that were also utilized used different measurements to illustrate pain and disability. Table 2 demonstrated some characteristics of the included studies. Firstly, Jin et al. (27), Hallal (25), and Kapucu and Unver (29) classified similar pain grades as *Slight, Mild, Moderate, Severe, Extreme*. However, we were not able to synthesize the data together, owing to the fact that the data classification was not standardized. Secondly, the duration of the presence of pain was delved in researches of authors as Jin et al. (27), Hallal (25), and Ozdemir et al. (31). Jin et al. (27) research indicated that pain would continue for <2, 2–8, ≥8 week, respectively, 183 (51.1%), 116 (32.4%), 59 (16.5%). In Hallal (25), pain could be sever hour: 40 (47.6%), 1 day:11 (13.1%), several days: 11 (13.1%), several weeks: 4 (4.8%), constantly: 18 (21.4%). Ozdemir et al. (31) demonstrated 8.7 ± 5.27 year. Thirdly, NRS was used in Tulay et al. (37), Ribom et al. (33), and Scaturro et al. (35). Tulay et al. (37) focused on illustrating the pain duration. Ribom et al. (33) did not specify how low is the participants with fracture, which encountered difficulties in the comparison process. Scaturro et al. (35) study only mentioned that the pain (NRS) was significant if it was directly related to the number of vertebral fractures. Fourthly, EQ-5D was applied both by Ramírez-Pérez et al. (32) and Jung et al. (17). Ramírez-Pérez et al. (32) did not have the control group, resulting on the comparison not being successfully completed. Fifthly, Qualleffo-41 was used throughout the study of Fechtenbaum et al. (23) and Ciubean et al. (22) and provided also a different illustration, which we could not compare. Sixthly, there was a certain pain assessment used in the study: Von Korff Pain Intensity and Disability questionnaires (36), Geriatric Pain Scale (0–100) (29), Pain Regulation Questionnaire (PRQ) (13), Visual Analog Scale (VAS) (26). From the study of Jahelka et al. (26), which used the SF-36 and QUALEFFO, we could not access the pain domain solely and, thus. we did not include the data in Figure 3.

**Table 2:** Characteristics of included studies.

**References**	**Country**	**Duration**	**Participants**	**Female/male**	**Age(year)**	**Outcome (20, 21)**	**NOS score^a^**
Briem et al. (13)	Germany	Average follow-up of 5.3 ± 1.7 years after injury Range 3–8	Thoracolumbar fracture group = 85; control = 584	Thoracolumbar fracture group: 41/44 control: N/A	Thoracolumbar fracture group- Mean ± SD: 47.8 ± 12.8 Range 25–65 control: N/A	Thoracolumbar fracture vs. control group *(*Mean ± SD) *SF-36 questionnaire -pain domain 65.78 ± 2.87 vs. 78.90 ± 8.87 (*p* < 0.05) *Pain Regulation Questionnaire (PRQ) Pain competence: 41.40 ± 1.12 vs. 36.98 ± 8.41 (*p* < 0.001) Pain intensity: 23.84 ± 1.30 vs. 29.55 ± 8.62 (*p* < 0.001) Pain anxiety:26.63 ± 11.59 vs. 31.90 ± 8.58 (*p* < 0.001) Pain depression: 21.15 ± 1.27 vs. 25.33 ± 9.60 (*p* < 0.001) Pain avoidance: 25.72 ± 0.94 vs. 25.38 ± 8.10 Pain withdrawal: 24.93 ± 1.23 vs. 28.90 ± 10.90 (*p* < 0.001) Pain distraction: 33.29 ± 1.03 vs. 32.37 ± 8.21	S*** C** O***
Chou et al. (16)	Taiwan	Oct. 2002–Mar. 2003	24,435	11,937/12,498	Over 20 years	1.2,912 participants with osteoporosis, 1,416 reported low back pain (*p* < 0.001). 2. Osteoporosis vs. non- osteoporosis, with low back pain OR = 2.55 (95% CI = 2.33–2.78); with frequent low back pain OR = 4.15 (95% CI = 3.66–4.70) 3. Adjusted sociodemographic factors, ORs of associated osteoporosis or not for frequent low back pain in females and males were 3.49 (95% CI = 2.99–4.07) and 5.77 (95% CI = 4.66–7.15), respectively.	S**** C** O***
Ciubean et al. (22)	Romania	Jun. 2016–Aug. 2017	364 postmenopausal women	364/0	Mean ± SD: Osteoporosis (*n* = 228): 65.5 ± 7.39 Control (*n* = 136): 63.45 ± 8.16 Range: 46–85	*SF-36 questionnaire -pain domain [median (IQR)] 1. Osteoporosis vs. Control:45 (45; 67.5) vs. 72.5 (55; 77.5) (*p* < 0.001) 2. Osteoporosis patients with fracture (*n* = 132) vs. without fracture (*n* = 96): 45 (45; 67.5) vs. 45 (35; 57.5) (*p* = 0.035) *QUALEFFO-41 -pain domain [median (IQR)] 1. Osteoporosis patients with fracture (*n* = 132) vs. without fracture (*n* = 96): 55 (30; 65) vs. 50 (30; 65) (*p* = 0.446)	S** C* O***
Fechtenbaum et al. (23)	France	–	588 have osteoporosis	588/0	Mean ± SD vertebral fracture (*n* = 548) vs. control group: 71.61 ± 5.01 vs. 71.00 ± 5.13 (*p* = 0.43)	QUALEFFO scores- pain domain(0-100) patients with no fracture (*n* = 40): 60 patients with sum of grade of fracture is 1 or 2 (*n* = 133): 51 patients with sum of grade of fracture is 3 or 4 (*n* = 189): 58 patients with sum of grade of fracture is 5–9 (*n* = 146): 58 patients with sum of grade of fracture is ≥10 (*n* = 80): 55	S** C* O**
Finsen (24)	Norway	–	307 subjects age of 50 years	222/85	Over 50 years	Patients self-reported pain (Some gave more than one answer and horizontal aggregates of percentages are therefore >loo) None (no infirmity): 31 (10.1%); foot (foot/leg/knee pain): 135 (44%); back (back pain): 96 (31.3%); hip (hip pain): 53 (17.3%)	S** C* O**
Gheorghita et al. (4)	Canada	At least 1 year	67	55/12	Range: 47–89	34 participants reported pain (30 female,4 male).	S*** C* O***
Hallal (25)	USA	–	101 women with diagnosed postmenopausal osteoporosis	101/0	Mean: 62.6	1.84 participants reported the presence of back pain. 2. Frequency of back pain (daily: 33, weekly: 6, monthly: 20, less than once per month: 15) 3. Duration of back pain (sever hour: 40, 1 day: 11, several days:11, several weeks:4, constantly:18) 4.severity of back pain (very: 14, moderately: 45, mildly: 25)	S** C* O*
Jahelka et al. (26)	Austria	Jun 2007–Jun. 2008	222	173/49	Mean ± SD: total: 79.3 ± 8.5	Visual analog scale (0–10) Osteopenic patients: 3.2 ± 2.6 Osteoporotic patients without fracture history: 3.2 ± 2.5 Osteoporotic patients with fracture history: 3.9 ± 2.7 (*p* > 0.05)	S*** C** O***
Jin et al. (27)	China	Nov. 1, 2016–Sep. 30, 2018	358 with vertebral fractures	284/74	Mean ± SD: 72.3 ± 9.4	1. Pain duration, weeks (<2:183, 2–8:116, ≥8:59) 2. Spinal palpation tenderness: 197 3. Axial spinal percussion pain: 83 4. Radiating pain: 76 5. Pain grades (mild: 17, moderate: 121, severe: 220)	S*** C** O***
Jung et al. (17)	Korea	At least 6 month	196 with an osteoporotic vertebral compression fracture Reference population (28) = 600	Fracture group:165/31 Reference population:303/297	Mean ± SD: 72.7 ± 7.9	*EQ-5D (pain/discomfort domain) 1. No problem-39 (19.9%); 1 some problems: 139 (70.9%); serious problems: 18 (9.2%) 2. Age 50–59 (*n* = 13) vs. reference population 84.6 vs. 30.6% (*P* < 0.001) 3. Age ≥ 60 (*n* = 183) vs. Reference population 79.8 vs. 62.7% (*P* < 0.001)	S*** C* O**
Kapucu and Ünver (29)	Turkey	–	105 females with osteoporosis	105/0	Mean: 74.3 ± 7.5	Geriatric pain scale (0–100) 1. Mean: 57.6 ± 17.5; Min = 16.6; Max = 92.8 2. Pain level (*n* = 104) Slight (0–30) = 7 (6.7%); Mild (31–69) = 70 (67.3%); Severe (70–100) = 27 (26.0%)	S*** C* O**
Miyakoshi et al. (30)	JAPAN	–	174 consecutive women with postmenopausal osteoporosis	174/0	Mean ± SD back pain (*n* = 159) vs. Non-back pain (*n* = 15): 67.8 ± 6.5 vs. 65.5 ± 7.0 (*p* = 0.18)	1.159 patients (91.4%) complained of back pain.	S** C* O**
Qzdemir et al. (31)	Turkey	–	909 patients		Mean: 60 Range: 33–89	1.695 patients (76.45%) reported experiencing pain 2. The duration of the presence of pain was 8.7 ± 5.27 year [Min:1, Max: 26]	S** C* O**
Ramírez-Pérez et al. (32)	Mexico	6 month	136 with hip fracture	95/41	Mean ± SD: 77 ± 10	EQ-5D(pain/discomfort domain) 1. 1st, 3rd, and 6th month patients report pain, respectively, 122 (89.7%), 92 (68%), 72 (52.9%) 2. patients report pain, respectively, in level 1, 2, 3 1st month:148,735; 6th month: 646,210 (level 1: indicating no problem; level 2: indicating some problems; level 3: indicating extreme problems)	S** C** O***
Ribom et al. (33)	Sweden	–	36 women with osteoporosis and verified with vertebral fracture	36/0	Mean ± SD: 74.6 ± 8.3 Median: 76.6 Range: 57–87	Numeric rating scale (NRS) 1. Maximum pain: Mean ± SD: 5.9 ± 1.8; median: 6; range: 2–8 2. Minimum pain. Mean ± SD: 1.9 ± 2.5; median: 2; range: 0–8 3. Average pain: Mean ± SD: 4.8 ± 2.1; median: 5; range: 0–8	S** C* O***
Ross et al. (18)	USA	Each of ~1.5 years duration	1,098 Japanese ancestry	1,098/0	Mean: 63.3 Range: 43–80	*The original population (*n* = 1,098) 1. 200 of these women had responded to questions about back pain, the number who reported increased frequency of back pain after the fracture was 16 (46%) of 35 subjects with new vertebral fractures, 1 (10%) of 10 subjects with prevalent fractures only, and 21 (14%) of 155 subjects without vertebral fractures.	S*** C*
						2. Incidence of increased frequency of back pain With vertebral fractures vs. without vertebral fractures Incident fractures: OR = 6.4 (95% CI: 2.6–15.6); *p* < 0.05 Prevalent fractures OR = 1.7 (95% CI: 0.5–5.6): *p* > 0.05 *The most examination (*n* = 203) 1. 28.1% reported some frequency of back pain since their previous visit. 2. Among the subjects with and without incident vertebral fractures (*n* = 45 and 158), the proportions reporting some frequency of back pain were 53 and 21%, respectively.	O**
Sale et al. (34)	Canada	6 month	21 who had sustained fractures	16/5	Range: 51–87	11 participants reported persistent pain	S*** C* O***
Scaturro et al. (35)	Italy	Jan. 2016–Jan. 2018	513 post-menopausal women over 50, having back pain for at least 3 months, not responding to conservative treatment, with NRS between 2 and 4 (mild pain) and SF 36 between 60 and 100.	513/0	Mean: 72 Range: 50–89	Numeric rating scale (NRS) 1. 77.5 % (*n* = 165) of patients referred an NRS rate between 2 and 3 (first group) and 22.5% (*n* = 48) a rate of 4 (second group). 2. The correlation between the pain (NRS) and the number of vertebral fragility fractures (*P* < 0.001).	S** C* O**
Suzuki et al. (36)	Sweden	A year (Dec. 2003–Nov. 2006)	107	72/35	Mean ± SD: 75.5 ± 11.9 Range: 42–96	*Von Korff Pain Intensity score(0-100) 70.9 ± 19.3 (3 weeks), 61.5 ± 21.4 (3 months), 60.7 ± 21.6 (6 months), 60.5 ± 23.0 (12 months); *Von Korff Disability score (0–100) disability means scored 68.9 ± 23.6 (3 weeks), 56.4 ± 25.5 (3 months), 51.0 ± 27.5 (6 months), 53.9 ± 27.8 (12 months) (*P* < 0.001). *EQ-5D 1.Total score: 0.37 ± 0.37 (3 week), 0.52 ± 0.35 (3 months), 0.54 ± 0.36 (6 months), 0.52 ± 0.38 (12 month) (*p* < 0.001). 2. The number of patients reporting moderate or severe problems in pain/discomfort domain 97% (3 week), 89% (3 months), 87% (6 months), 89%(12 month) (*p* < 0.001).	S** C* O**
Tulay et al. (37)	Turkey	Jan.-Dec. 2016	172 with rib fracture	66/106	Medican: 47 Range: 18–85	Numeric rating scale (NRS) (0–10) 1. At 15th days, 3rd month, 6th month, the pain level of <65 yr participants were significant lower than ≥65 yrs group. 2. At 15th days, 3rd month, 6th month, the pain level of < female were significant higher than ≥ male. 3. Patients have 2 rib fractures with significant higher pain level than who has only one fracture.	S** C* O***
Zetterberg et al. (19)	Sweden	1 year	Hip fracture patients:868 (final was 840 patients) Control group: 2,251	Hip fracture patients: 623/245 Control group: 1,333/918	Hip fracture patients mean Female: 79.0 Male: 73.9	Back pain during last 10 years 1. Female (*P* < 0.001) Hip fracture patients: 23% (*n* = 143) vs. Control group: 45% (*n* = 600) 2. Male (*P* < 0.001) Hip fracture patients:20% (*n* = 49) vs. Control group: 48% (*n* = 441)	S** C* O**

a Scale domains: S selection of study groups, C comparability, O outcome assessment. Each “*” counts one point in different domain.

#### Self-Reported Pain

In 10 studies have patients self-reported pain (4, 16, 18, 23–25, 27, 30, 31, 34). Chou et al. (16) study had 2,912 participants from which 1,416 reported some kind of low back pain (48.6%, *p* < 0.001). Fechtenbaum et al. (23) 548 of 588 reported pain problems. Finsen (24) could report 276 cases of pain out of 307 subjects who had suffered any kind of fracture, among the 307 participants who reported pain, the different body parts affected would be seen as followed, foot (foot/leg/knee pain):135 (44%); back (back pain): 96 (31.3%); hip (hip pain):53 (17.3%). Gheorghita et al. (4) enrolled 67 applicants who had suffered from any kind of fracture, from which 34 of them reported fracture-related pain (5.7%) ith fragility fracture. During Hallal's (25) research, 84 participants (83.1% out of the total) reported some presence of back pain. Jin et al. (27) reported 197 (55.0%) spinal palpation tenderness, 82 (23.2%) axial spinal percussion pain, and 76 (21.2%) radiating pain. Miyakoshi et al. (30) reported 159 patients (91.4%) who would show some kind of discomfort related to back pain. Ozdemir et al. (31) showed 695 patients (76.45%) who had reported experiencing pain. Ross et al. (18), 28.1% (*n* = 203) reported frequent back pain in the studied patients. Sale et al. (34) recruited 21 participants whose ages ranged from 51 to 87, from which 11 individuals self-reported constant pain after a fracture. They also reported movement-related limitations including difficulties related to a range of motion, lifting capacity, or insufficient strength. On the other hand, the other 10 participants reported not suffering pain at the site of fracture. Similarly, there were four of them who reported a limited range of motion.

### Risk of Bias Assessments

Twenty-one of the included studies were rated on the NOS scale, where the higher the score rated the better quality it would proof (see Table 2), a total of 7 were rated as “high quality,” and none of the included studies was rated as “low quality.” Also, ROBINS-I was used to evaluate the risk of bias throughout the study (Table 3). Six articles were evaluated as “moderate risk” and 1 as “high risk” to have bias. Due to the small number of papers and the degree of heterogeneity in study designs, interventions, and outcome indices, the meta-analysis was not considered fully appropriate.

**Table 3:** Risk of bias assessment using ROBINS-I.

**References**	**Type of research**	**Pre-intervention**		**At intervention bias in classification of intervention**	**Post-intervention**				**Total**
		**Bias due to confounding**	**Bias in selection participants into study**		**Bias due to deviations from intended interventions**	**Bias due to missing data**	**Bias in measurement of outcomes**	**Bias in selection of the reported outcomes**	**Total bias**
Briem et al. (13)	Retrospective	Low	Low	Low	Low	Low	Low	Low	Low
Chou et al. (16)	Cross-sectional	Low	Low	Low	Low	Low	Moderate	Moderate	Moderate
Ciubean et al. (22)	Cross-sectional	Low	Low	Low	Low	Low	Low	Low	Low
Fechtenbaum et al. (23)	Prospective	Low	Low	Low	Low	Low	Low	Moderate	Low
Finsen (24)	Quantitative cohort	Low	Low	Low	Low	Moderate	Moderate	Moderate	High
Gheorghita et al. (4)	Qualitative cohort	Low	Low	Unclear	Low	Low	Moderate	Moderate	Moderate
Hallal (25)	Prospective	Low	Low	Low	Low	Low	Moderate	Moderate	Moderate
Jahelka et al. (26)	Prospective	Low	Low	Low	Low	Low	Low	Low	Low
Jin et al. (27)	Prospective	Low	Low	Low	Low	Low	Low	Moderate	Low
Jung et al. (17)	Ambispective	Low	Low	Low	Low	Low	Low	Low	Low
Kapucu and Ünver (29)	Descriptive	Low	Low	Low	Low	Low	Low	Low	Low
Miyakoshi et al. (30)	Observational	Low	Low	Low	Low	Low	Low	Moderate	Low
Qzdemir et al. (31)	Retrospective	Low	Low	Low	Low	Low	Low	Moderate	Low
Ramírez-Pérez et al. (32)	Prospective	Low	Low	Low	Low	Moderate	Low	Low	Low
Ribom et al. (33)	Prospective	Low	Low	Low	Low	Moderate	Low	Moderate	Moderate
Ross et al. (18)	Cross-sectional	Low	Low	Low	Low	Low	Low	Moderate	Low
Sale et al. (34)	Qualitative cohort	Low	Low	Unclear	Low	Low	Moderate	Moderate	Moderate
Scaturro et al. (35)	Observational	Low	Low	Low	Low	Low	Low	Low	Low
Suzuki et al. (36)	Prospective cohort	Low	Low	Low	Low	Low	Low	Moderate	Low
Tulay et al. (37)	Prospective	Low	Low	Low	Low	Low	Low	Low	Low
Zetterberg et al. (19)	Prospective	Low	Low	Low	Low	Moderate	Moderate	Low	Moderate

### The Result Summary of ROBIS

A summary of the findings and the ROBIS assessment for each domain can be seen in Table 4. In phase 2, study eligibility criteria, identification and selection of studies, data collection, and study appraisal were rated as “low risk.” Due to the fact that the used studies included different assessments, synthesis, and findings, the domain was rated as “high risk.” Throughout phase 3, the overall risk of bias was rated “low risk.”

**Table 4:** Risk of Bias in Systematic Reviews (ROBIS tool) of the study.

**Phase 2**				**Phase 3**
**Study eligibility criteria**	**Identification and selection of studies**	**Data collection and study appraisal**	**Synthesis and findings**	**Risk of bias in review**
Low risk	Low risk	Low risk	High risk	Low risk

^*^Possible risk of bias levels: low, high, unclear.

## Discussion

### Clinical Implications

To the best of our knowledge, we comprehend this study to be the first systematic review and meta-analysis to examine the impacts of pain among the fragility fracture population. Our study results support the hypothesis that frail patients with fractures were suffering from a continuous risk of pain, and as this further exceeded the typical length of time assumed essential for curing and resolution of pain. Also, our results also provide more clear evidence related to patients undergoing fragility fracture may experience significant long-term pain effects.

The trajectories of frailty degrees in the elder population could vary substantially, particularly when estimating the short-term or long-term treatment effect, personal lifestyle change, related comorbidity development, and severe disease progression (38). Exploring the frailty transition chronologically would help clinicians to obtain a further knowledge related to the effects modifications of frailty and a better estimate on future fracture risk. Previous studies indicated that due to changes in the spine shape and height loss, patients may experience uninterrupted back pain even after the acutely painful episode subsides after a vertebral fracture (4, 39). Wrist fractures also have been projected to be able to recover after 6 weeks' post-fracture (40). However, another study showed one physiological cycle of bone which would remodel in healthy adults, lasting from 4 to 6 months (41). Risk fracture is a most frequent feature on the Complex regional pain syndrome(CRPS) (42). Patients with hip fractures often presented comorbidities and cognitive impairment that frequently prevented their recovery (43).

To examine the relationship between fragility fracture and pain, our study included only observational studies. Ordinary meta-analyses on the efficacy of interventions merely obtained high-quality evidence from randomized controlled trials (6). However, randomized controlled trials are often not the best source of evidence for harm due to the study duration is often too short to detect long-term or rare adverse events (44, 45). In addition, it is not possible to randomize patients into the categories “with fragility fracture” or “without fragility fracture.” Including observational studies in this systematic review was a strong point, as these studies could indicate the effect of short-term and long-term pain in the fragility fracture population.

#### Clinical Practice

This systematic review found that there is an influence of pain following fragility fracture. Based on the results, medical teams should develop the treatment and rehabilitation protocol to prevent or reduce the pain of post-fracture, and the protocol should include meditation, exercises, and integrated physical treatment. Some consideration about the role of pain killers as well as anti-osteoporosis drugs for pain relief in fragility fracture patients should be provided (46–48). For better pain improvement, the program should be continuous, progressive, and combined alternative strategies.

### Methodological Considerations

From the methodological viewpoint, our study included several limitations. Firstly, based on the current information, we could not assess the fluctuating frailty status concerning the risk of fragility fracture. Secondly, due to the number of selected studies that could be quantified, it was not sufficient. Due to the various measurements, it was difficult to conduct a meta-analysis with enough sample sizes. Finally, when we used the ROBIS approach to assess the quality of the evidence for the systematic review, the evidence from all the included observational studies was initially rated as relatively low quality because of imprecision. The addition of more studies in the future may increase the quality of evidence.

### Conclusions

The current evidence could not fully support that pain continues to influence patients' lives after a fragility fracture. However, it still exposed the pain might come with fracture. The findings also could be useful to help health care providers to better recognize and manage this clinical consequence of fractures. We recommend research on a wider range of populations to provide more comprehensive and accurate findings.

## Data Availability Statement

The raw data supporting the conclusions of this article will be made available by the authors, without undue reservation.

## Author Contributions

P-EC: conceptualization, methodology, software, data curation, and writing-original draft preparation. T-HT: writing-reviewing and editing. C-WC: supervision. All authors contributed to the article and approved the submitted version.

## Conflict of Interest

The authors declare that the research was conducted in the absence of any commercial or financial relationships that could be construed as a potential conflict of interest.
